# Autochthonous Probiotics Alleviate the Adverse Effects of Dietary Histamine in Juvenile Grouper (*Epinephelus coioides*)

**DOI:** 10.3389/fmicb.2021.792718

**Published:** 2021-12-07

**Authors:** Zi-Yan Liu, Hong-Ling Yang, Ling-Hao Hu, Wei Yang, Chun-Xiang Ai, Yun-Zhang Sun

**Affiliations:** ^1^Xiamen Key Laboratory for Feed Quality Testing and Safety Evaluation, Fisheries College, Jimei University, Xiamen, China; ^2^Xiamen Jiakang feed Co., Ltd, Xiamen, China; ^3^College of Ocean and Earth Science, Xiamen University, Xiamen, China

**Keywords:** autochthonous probiotics, histamine, growth performance, innate immunity, gut health, *Epinephelus coioides*

## Abstract

High dose (0.3%) of dietary histamine can cause adverse effects on growth performance, innate immunity, and gut health in juvenile grouper (*Epinephelus coioides*). In the present study, three autochthonous probiotics (*Bacillus pumilus* SE5, *Psychrobacter* sp. SE6, and *Bacillus clausii* DE5) were supplemented separately to diets containing 0.3% of histamine and their effects on growth performance, innate immunity, and gut health of grouper (*E. coioides*) were evaluated in a 56-day feeding trial. The results showed considerable increase in weight gain, specific growth rate, hepatosomatic index, and decreased feed conversion rate in groupers fed with probiotic-supplemented diets. Supplementation of autochthonous probiotics has improved antioxidant capacity and innate immunity of *E. coioides* by measuring correlative parameters, such as total antioxidant capacity, superoxide dismutase activity, malondialdehyde content, and so on. Additionally, dietary probiotics have significantly reduced the levels of serum interleukin-1β (at days 28 and 56), fatty acid-binding protein 2, and intestinal trefoil factor (at day 28), and promoted intestinal integrity following remarkably increased muscle thickness and mucosal fold height at day 56, especially in grouper fed with *B. pumilus* SE5 containing diet (*P* < 0.05). On day 56, the gut microbial composition of *E. coioides* was positively shaped by autochthonous probiotics, the relative abundance of potentially pathogenic *Photobacterium* decreased while beneficial *Lactobacillus* increased in fish fed with probiotic strains, especially with *B. pumilus* SE5 and *B. clausii* DE5. These results suggest that among the three autochthonous probiotic strains tested, *B. pumilus* SE5 is showing better efficiency in alleviating the adverse effects of (high levels) dietary histamine by decreasing the expression of inflammatory markers and by improving the growth, innate immunity, and gut health of juvenile grouper *E. coioides*.

## Introduction

Recently, owing to limitation of global supply and increase in demand of white fish meal (WFM), the traditional and preferred source of animal protein ingredient for commercial aquafeeds, brown fish meal (BFM) has served as a common substitute for WFM. However, it is reported that BFM contains more histamine than WFM (Zhai et al., 2020). Histamine is one of the most common biogenic amines found in fish meal and is derived from decarboxylation of corresponding free histidine by microorganisms (e.g., *Photobacterium*, *Enterobacter*, etc.) (Visciano et al., 2020). High doses of histamine in aquatic feed can cause adverse effects on growth performance, innate immunity, and/or gut health in grouper, *Epinephelus coioides* (Liu et al., 2021), American eel, *Anguilla rostrata* (Zhai et al., 2020), yellow catfish, *Pelteobagrus fulvidraco* (Li et al., 2018), Chinese mitten crab, *Eriocheir sinensis* (Zhao et al., 2012, 2016), and blue shrimp, *Litopenaeus stylirostris* (Tapia-Salazar et al., 2001). The negative impacts of histamine dosage greatly affect the aquaculture industry, causing major economic loss, hence alleviating its negative impacts in the aquatic animals is crucial by employing economic and effective dietary strategies.

Extensive research has shown that probiotics can alleviate inflammation, positively shape the gut microbiota, facilitate growth performance, and boost the innate immunity in aquatic animals (Liu et al., 2009; Gobi et al., 2018; Xia et al., 2018; Dawood, 2021; Yeganeh et al., 2021). Recently, many researchers have focused on the application of autochthonous probiotics in aquaculture (Sun et al., 2013, 2014; Talpur et al., 2013; Doan et al., 2018; Feng et al., 2019; Liu et al., 2020; Tang et al., 2020; Liao et al., 2021), owing to there is a general consensus that ideal probiotics should be derived from a host or a host environment. A key advantage is that autochthonous probiotics can efficiently colonize, multiply, and work effectively in the host intestine (Lazado et al., 2015). However, it is still not known whether autochthonous probiotics can alleviate the negative effects of dietary histamine in marine fish.

Groupers (*Epinephelus* sp.) have been widely bred in South-East Asia because of their advantages of high consumer demand, fast growth, and high nutritional and economic value. The annual yield of groupers was reported to be about 192,045 tons in 2020 (China Fishery Statistics Yearbook, 2021). Based on the performance analysis studied previously (Sun et al., 2009), *Bacillus pumilus* SE5, *Psychrobacter* sp. SE6, and *Bacillus clausii* DE5 (GenBank accession numbers EU520340, EU520334, and EU520331) isolated from the gut of the grouper was selected for this study. The performance analysis of the three autochthonous probiotics includes: (1) *in vitro* analysis showed profound inhibitory ability against several fish pathogens and considerable tolerance to artificial gastric and intestinal fluids, and (2) *in vivo* analysis showed that the application of three probiotic strains (1.0 × 10^8^ CFU/g diet) to grouper (*E. coioides*) facilitates its growth by enhancing innate immunity and maintaining intestinal microbiota (Sun et al., 2013, 2014). Our recent study has demonstrated that 0.3% dietary histamine can suppress growth, decrease innate immunity, and cause inflammation and severe gut injury in *E. coioides* (Liu et al., 2021). The gut microbiota is strongly associated with host health and well-being, and probiotics can positively shape and maintain the gut microbial balance (Hoseinifar et al., 2018), in this study we have determined the effects of the three autochthonous probiotic strains on groupers’ health and gut microbiota. Briefly, the impact of these probiotic strains on the growth, immunity, inflammation, gut morphology, and microbiota were investigated in the groupers’ fed with 0.3% dietary histamine. Supplementation of the diet with 0.3% histamine enables us to evaluate whether these autochthonous probiotics can alleviate the adverse effects of high doses of dietary histamine in juvenile grouper (*E. coioides*).

## Materials and Methods

### Probiotic Strains

Three autochthonous probiotics (*B. pumilus* SE5, *Psychrobacter* sp. SE6, and *B. clausii* DE5) isolated from the gut of the grouper were selected for this study. The culture media and growth conditions were maintained according to the methods of Sun et al. (2009, 2011).

### Diet Preparation

Based on our previous study, supplemented with 0.3% histamine to a basal diet exerted significantly negative impact on grouper (Liu et al., 2021). Therefore, this diet (a basal diet with 0.3% histamine) was set as the control diet (T1) in this study. All dietary ingredients were mixed thoroughly in an electric mixer, blended with the supplementation of oil source and water with 0.3% histamine to form a paste, which was then passed through a two-screw extruder equipped with a 2.5-mm die to obtain uniform pellets, then probiotic strains were sprayed to the surface of the control diet at the level of 1.0 × 10^8^ CFU/g (diets T2–T4, Table 1). Finally, all experimental diets were air dried under sterile conditions and then stored at 4°C to maintain dietary quality. To keep up the viability of probiotics, new diets were produced every 4 weeks with reference to our previous study (Sun et al., 2014).

**TABLE 1:** The test groups and diets.

Test groups	Diet
**Control group**	
T1	Control diet (a basal diet supplemented with 0.3% histamine)
**Experimental groups**	
T2	Control diet + probiotic *B. pumilus* SE5
T3	Control diet + probiotic *Psychrobacter* sp. SE6
T4	Control diet + probiotic *B. clausii* DE5.

### Animals and Experimental Conditions

Healthy juvenile groupers (*E. coioides*) were purchased from the Haikang Aquaculture Research Base of Dabeilong Aquaculture Group (Zhangzhou, China). Before the test experiment, the groupers (*E. coioides*) were allowed to adapt to the laboratory environment by feeding with basal diet for a period of 14 days. After adaptation, 480 juveniles (average initial weight: 21 ± 0.41 g) were divided into 16 tanks (supplementing 300 L seawater, salinity: 30–32 g/L), i.e., 30 fish per tank in quadruplicate based on random allocation principle. Fish were hand-fed daily at 08:00 a.m. and 18:00 p.m. to apparent satiation during the whole feeding trial, and three-fifths of the seawater was changed daily in the re-circulating aquaculture system. The rearing water quality was monitored daily and maintained approximately stable (temperature: 18–26°C, pH: 7.5–8.2, dissolved oxygen: ≥7.5 mg/L, and ammonia nitrogen levels: <0.2 mg/L).

### Experimental Design

After overnight starvation, 10 fish were randomly collected from each tank (four tanks per group), thus 40 fish for each group were collected to evaluate the growth performance at days 28 and 56. The collected fish were anesthetized batch wise by using eugenol (100 ppm), and each of the fish in all the tanks were weighed for analyzing the growth performance and survival rate. Blood was collected from the caudal vein of the fish, quickly transferred to 1.5 mL sterile tube and stored at 4°C for 12 h. Then, serum was separated and pooled following centrifugation at 10,000 rpm at 4°C for 10 min and stored in 1.5 mL Eppendorf tubes at −80°C for subsequent analysis. Liver and intestine samples were aseptically removed, weighed individually, and stored at −80°C for further analysis. Foreguts were collected from four fish from each tank by random sampling and the samples were kept in Bouin’s fixative solution (PH0976; Phygene Life Sciences Company, Fuzhou, China) for less than 24 h (completely immersed) for histomorphological evaluation. All the above samples were collected on days 28 and 56. At day 56, whole intestine (four samples per group) was sampled to evaluate intestinal microbiota.

### Measurement of Serum and Liver Biochemical Parameters

Serum total antioxidant capacity (T-AOC) and activities of alkaline phosphatase (AKP), acid phosphatase (ACP), and superoxide dismutase (SOD) were detected spectrophotometrically by using commercial kits (Nanjing Jiancheng Bioengineering Institute, Nanjing, China) following the manufacturer’s protocol.

The liver samples were homogenized with ice-cold 0.01 M phosphate buffered saline (PBS, pH: 7.2–7.4; *w:v* = 1:9), centrifuged at 3000 rpm at 4°C for 10 min and the supernatant was collected. Malondialdehyde (MDA, Beijing Solarbio Science & Technology Co., Ltd.) concentration and activities of glutamic oxaloacetic transaminase (GOT) and glutamic propylic transaminase (GPT) of the supernatant were measured by using commercial kits (Nanjing Jiancheng Bioengineering Institute, Nanjing, China).

### Serum and Intestinal Inflammatory Factors

Levels of serum fatty acid-binding protein 2 (FABP2) were detected following the instruction of the enzyme-linked immunosorbent assay (ELISA) kit (Nanjing Jiancheng Bioengineering Institute, Nanjing, China). ELISA kits for determination of interleukin 1 beta (IL-1β), serum amyloid A (SAA), and C-reactive protein (CRP) contents in serum were provided by Shanghai Jianglai Biotechnology Co., Ltd. (Shanghai, China).

Gut samples were rinsed in ice-cold PBS [gut weight (g): PBS volume (mL) = 1:9], subsequently homogenized and centrifuged at 3000 rpm for 10 min at 4°C. The protein concentration of the supernatants were determined by using BCA Protein Assay Kit (Beijing LABLEAD, Inc., Beijing, China). ELISA Kit (Shanghai Jianglai Biotechnology Co., Ltd., Shanghai, China) was used to measure the intestinal trefoil factor (ITF) in the supernatant. The ITF levels were expressed in pg/mg protein.

### Gut Morphology

Gut morphology was evaluated by preparing hematoxylin and eosin (HE) stained sections according to Liu et al. (2021). In brief, four pre-fixed foregut samples in each group were dehydrated with varying concentrations of ethanol and xylene treatment. Then, about 1.0 cm foregut were embedded in paraffin, and cut into 6 μm thin slices. Finally, the thin slices were stained with HE. Microscopic observation was performed by using Leica DM5500B Microscope (Germany). Meanwhile, muscular thickness (MT) and mucosal fold height (MFH) were determined by using Lecia Application Suite Version 4.7.0 (Leica Microsystems, Germany).

### Gut Microbiota

Quadruplicate gut samples per group were taken to perform high-throughput sequencing at day 56. Bacterial DNA of four gut tissue samples from each test group was extracted by using a DNA extraction kit (Beijing Solarbio Science & Technology Co., Ltd., China). The quantity and purity of the DNA were assessed by using a Nano-Drop^®^ND-1000 spectrophotometer (Nano-Drop Technologies, Wilmington, DE, United States). The integrity of the extracted genomic DNA was determined by electrophoresis on a 1.2% (w/v) agarose gel. Then, the V3 + V4 hypervariable regions of the 16S rRNA gene of gut bacteria were amplified with the forward primer 338F (5′-ACTCCTACGGGAGGCAGCA-3′) and the reverse primer 806R (5′-GGACTACHVGGGTWTCTAAT-3′) by using polymerase chain reaction (PCR) method. Purified amplification products were transferred to Beijing Biomarker Biotechnology Co. Ltd. (Beijing, China). The products were sequenced using the Illumina HiSeq 2500 platform. The sequencing data of all samples have been uploaded to Sequence Read Archive (SRA) under BioProject ID PRJNA729733. Raw tags were filtered using Trimmomatic (v0.33) and UCHIME (v4.2) in order to remove the low-quality sequences and chimeras. Then, sequences with ≥97% similarity were assigned to the same operational taxonomic units (OTUs) using UCLUST in QIIME V1.8.0 package. A representative sequence for each OTU was annotated by searching the SILVA database^1^. The unique and shared OTUs were displayed as a Venn diagram. We performed the gut microbiota community analysis on the BMKCloud platform^2^.

### Calculations and Statistical Analysis

Different parameters of growth performances and feed utilization were calculated through the following equations.

Weightgainrate(WGR,%)=100×(finalbodyweight⁢-initialbodyweight)/initialbodyweight

Specificgrowthrate(SGR,%/d)=100×(lnfinalbodyweight⁢-lninitialbodyweight)/daysoffeedingtrial

Feedconversionrate(FCR)=feedintake/(finalbodyweight⁢-initialbodyweight)

Hepatosomaticindex⁢(HSI,%)=100×(liver⁢weight/body⁢weight)

Survivalrate⁢(SR,%)=100×number⁢of⁢survived⁢fish⁢in⁢sampling/30.

All the data obtained from treatment experiments were subjected to one-way ANOVA, Duncan’s multiple comparison test, and the statistical significance estimations were performed by using SPSS statistical software v.26.0 (IBM, Armonk, NY, United States). The statistical differences were documented as significant when *P*-value < 0.05 and were expressed as exhibited as means ± SE.

## Results

### Growth Performance and Feed Utilization

Grouper fed diets with/without autochthonous probiotics were recorded with SR above 97.5% and there was no significant variation among control (T1) and experimental groups (T2, T3, and T4) (*P* > 0.05) (Table 2) at the initial feeding period (days 0–28) or the whole feeding period (days 0–56). Further, dietary probiotic supplementation (Groups T2, T3, and T4) has improved WGR, SGR of *E. coioides* on days 28 and 56 without showing any significant statistical difference (*P* > 0.05). The FCR in the probiotic supplemented groups (T2, T3, and T4) was low as compared to the control group (T1) at days 28 and 56, and there was significant decrease in FCR observed only on day 28 (*P* < 0.05). HSI of *E. coioides* was not found to be affected by dietary supplementation of the probiotic bacteria on both days 28 and 56, showing no statistical differences in the values (*P* > 0.05).

**TABLE 2:** Effects of autochthonous probiotics on growth performance of grouper (*E. coioides*) fed diets with high level of histamine.

		Control group	Experimental groups		
		T1	T2	T3	T4
0−28 days	WGR (%)	78.33 ± 9.43	85.58 ± 8.87	84.66 ± 12.08	82.26 ± 4.45
	SGR (%)	2.21 ± 0.20	2.36 ± 0.18	2.34 ± 0.24	2.31 ± 0.09
	FCR	1.26 ± 0.01^a^	1.15 ± 0.02^b^	1.16 ± 0.01^b^	1.17 ± 0.01^b^
	HSI	1.48 ± 0.11	1.73 ± 0.15	1.65 ± 0.09	1.51 ± 0.08
	SR (%)	99.17 ± 0.83	100.00 ± 0.00	98.33 ± 0.96	99.17 ± 0.83
0−56 days	WGR (%)	84.67 ± 9.13	115.35 ± 12.68	100.00 ± 15.51	102.81 ± 11.30
	SGR (%)	1.09 ± 0.09	1.37 ± 0.11	1.22 ± 0.14	1.25 ± 0.10
	FCR	1.28 ± 0.08	1.10 ± 0.09	1.16 ± 0.12	1.20 ± 0.12
	HSI	2.08 ± 0.10	2.56 ± 0.28	2.22 ± 0.11	2.51 ± 0.14
	SR (%)	97.50 ± 1.60	98.33 ± 0.96	98.33 ± 0.96	98.33 ± 1.67

*Different characters in the same row data indicate significant differences (P < 0.05).*

### Serum Innate Immune Index

The serum ACP, AKP, T-AOC, and SOD activities of *E. coioides* demonstrated no significant difference among all tested groups (control – T1 and experimental groups T2, T3, and T4) on day 28 (*P* > 0.05) (Table 3). On day 56, the activities of ACP, AKP, T-AOC, and SOD in the serum of all the experimental groups (except T2) were found to be slightly increased, but with no statistical significance as compared to those of the control group (T1), though group T2 (groupers fed with diet containing *B. pumilus* SE5 – 1.0 × 10^8^ CFU/g diet) showed considerable variations in the values.

**TABLE 3:** Effects of autochthonous probiotics on serum immune index of grouper (*E. coioides*) fed diets with high levels of histamine.

		Control group	Experimental groups		
		T1	T2	T3	T4
28 days	ACP (U/100 mL)	4.60 ± 0.43	4.78 ± 0.64	4.89 ± 0.77	5.01 ± 0.63
	AKP (U/100 mL)	15.84 ± 1.58	18.58 ± 0.51	16.40 ± 1.68	15.99 ± 1.37
	T-AOC (mM)	1.12 ± 0.11	1.15 ± 0.11	1.25 ± 0.09	1.21 ± 0.08
	SOD (U/mL)	122.18 ± 19.18	126.57 ± 12.82	138.79 ± 10.77	131.00 ± 12.92
56 days	ACP (U/100 mL)	3.79 ± 0.73	5.06 ± 0.70	4.81 ± 0.89	4.07 ± 0.62
	AKP (U/100 mL)	9.35 ± 1.37	11.77 ± 1.70	11.62 ± 1.97	10.25 ± 0.63
	T-AOC (mM)	0.75 ± 0.02	0.80 ± 0.04	0.77 ± 0.05	0.80 ± 0.03
	SOD (U/mL)	112.04 ± 9.66	134.61 ± 17.27	121.02 ± 1.58	116.79 ± 10.23

*ACP, acid phosphatase; AKP, activities of alkaline phosphatase; T-AOC, total antioxidant capacity; SOD, superoxide dismutase.*

### Liver Biochemical Parameters

The liver biochemical parameters of *E. coioides* fed test diets are shown in Table 4. Dietary supplementation of three autochthonous probiotics in the diets of groupers (Experimental groups – T2, T3, and T4) showed significant decrease in liver MDA levels on day 28 (*P* < 0.05). Whereas the activities of GOT and GPT in all experimental groups (T2, T3, and T4) were higher than those in the control group (T1), especially groups T2 and T4 showed significant improvements in GOT and GPT activities (*P* < 0.05). On day 56, the MDA levels were again found to decrease and the GOT and GPT activities were found to increase, but the differences were not statistically significant.

**TABLE 4:** Effects of autochthonous probiotics on liver biochemical indices of grouper (*E. coioides*) fed diets with high levels of histamine.

		Control group	Experimental groups		
		T1	T2	T3	T4
28 days	MDA (nmol/gprot)	8.75 ± 0.41^a^	7.07 ± 0.65^b^	6.12 ± 0.63^b^	6.21 ± 0.43^b^
	GOT (U/gprot)	34.52 ± 6.53^a^	57.86 ± 3.56^b^	46.98 ± 8.15^ab^	59.55 ± 9.12^b^
	GPT (U/gprot)	54.38 ± 4.13^a^	70.45 ± 4.76^b^	67.67 ± 2.00^ab^	66.28 ± 5.09^ab^
56 days	MDA (nmol/gprot)	8.17 ± 0.96	6.22 ± 0.78	6.09 ± 0.37	6.10 ± 0.21
	GOT (U/gprot)	33.97 ± 2.61	41.78 ± 7.11	36.62 ± 2.53	50.69 ± 8.22
	GPT (U/gprot)	69.84 ± 1.33	76.54 ± 2.33	76.63 ± 4.83	78.89 ± 3.70

*Different characters in the same row data indicate significant differences (P < 0.05).*

*MDA, malondialdehyde; GOT, glutamic oxaloacetic transaminase; GPT, glutamic propylic transaminase.*

### Serum and Gut Inflammation Markers

Dietary supplementation of autochthonous probiotics did not significantly alter serum concentrations of SAA and CRP (Table 5) present in *E. coioides* both on days 28 and 56 (*P* > 0.05). After 28 and 56 days of feeding, a significantly lower serum IL-1β concentration was found in experimental groups (T2, T3, and T4) than the control group (*P* < 0.05). The levels of serum FABP2 and ITF on day 28 were found to decrease remarkably in groups T2 and T3 as compared to the control (*P* < 0.05). On day 56, the contents of serum FABP2 and intestinal ITF in all experimental groups (T2, T3, and T4) were found to be lower than the control group, although the results did not present significant difference (*P* > 0.05). Groups T2 and T3 showed almost similar levels of FABP2 and ITF, while the lowest levels of serum FABP2 and intestinal ITF were found in the T2 group (fish fed with a diet containing *B. pumilus* SE5).

**TABLE 5:** Effects of autochthonous probiotics on inflammation markers of grouper (*E. coioides*) fed diets with high levels of histamine.

		Control group	Experimental groups		
		T1	T2	T3	T4
28 days	CRP (μg/mL)	11.95 ± 0.92	8.79 ± 0.99	9.40 ± 1.45	8.01 ± 1.70
	SAA (μg/mL)	10.11 ± 1.35	7.92 ± 0.56	7.42 ± 1.34	7.45 ± 1.00
	IL-1β (ng/L)	83.11 ± 2.68^a^	70.58 ± 5.06^b^	69.01 ± 9.34^b^	63.40 ± 7.65^b^
	FABP2 (ng/mL)	17.15 ± 2.77^a^	10.64 ± 1.49^b^	10.04 ± 1.02^b^	15.60 ± 2.87^ab^
	ITF (pg/mgprot)	245.10 ± 27.42^a^	138.57 ± 14.76^b^	150.83 ± 11.14^b^	188.17 ± 33.29^ab^
56 days	CRP (μg/mL)	6.59 ± 1.08	4.91 ± 1.05	4.67 ± 1.00	4.44 ± 0.96
	SAA (μg/mL)	6.61 ± 1.15	6.11 ± 0.51	5.81 ± 0.34	6.06 ± 0.49
	IL-1β (ng/L)	92.55 ± 10.47a	71.49 ± 7.12b	81.26 ± 3.02b	75.80 ± 10.97b
	FABP2 (ng/mL)	19.11 ± 2.37	16.71 ± 1.02	17.78 ± 2.49	17.45 ± 1.59
	ITF (pg/mgprot)	238.45 ± 37.75	181.79 ± 15.77	193.10 ± 22.36	211.83 ± 25.83

*Different characters in the same row data indicate significant differences (P < 0.05).*

*IL-1β, interleukin-1 beta; SAA, serum amyloid A; CRP, C-reactive protein; ITF, intestinal trefoil factor; FABP2, fatty acid-binding protein 2.*

### Gut Morphology

**FIGURE 1:**
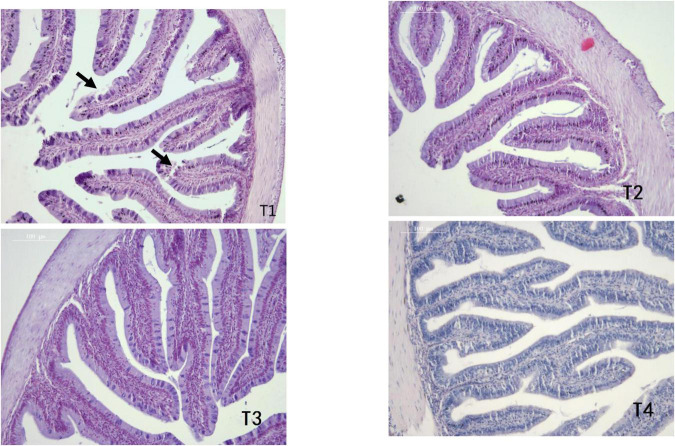
Photomicrographs of transverse HE-stained sections of the foregut of *E. coioides* fed tested diets for 28 days (100×).

**FIGURE 2:**
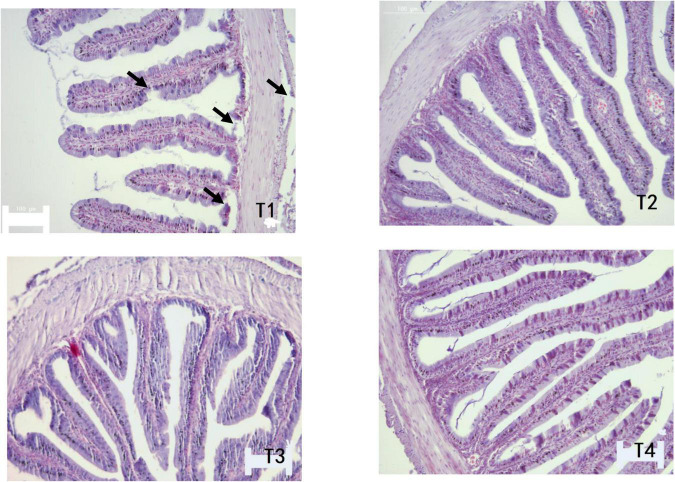
Photomicrographs of transverse HE-stained sections of the foregut of *E. coioides* fed tested diets for 56 days (100×).

As shown in Table 6, MT and MFH in all experimental groups (T2, T3, and T4) were found to be higher than the control group on day 28. On day 56, the MT and MFH in all experimental groups (T2, T3, and T4) were significantly higher than that of the control group, especially in group T2 (*P* < 0.05). The gut section of the control group (T1) showed symptoms of damage, mainly reflected as an inferior MT and MFH on day 28, further the more serious damage was observed in the control group (T1) on day 56 as indicated by arrows in Figures 1, 2. Inspiringly, relatively well-developed gut morphology was observed in groupers fed probiotic supplemented diets (Figures 1, 2).

**TABLE 6:** Effects of autochthonous probiotics on anterior intestinal morphology of grouper (*E. coioides*) fed diets with high levels of histamine.

		Control group	Experimental groups		
		T1	T2	T3	T4
28 days	MT (μm)	72.77 ± 5.74	76.01 ± 4.91	86.96 ± 3.80	82.78 ± 5.45
	MFH (μm)	210.28 ± 11.49	223.48 ± 11.06	235.29 ± 9.84	229.66 ± 9.63
56 days	MT (μm)	70.93 ± 1.17^c^	90.41 ± 2.62^a^	77.35 ± 3.30^bc^	83.03 ± 1.72^b^
	MFH (μm)	205.12 ± 8.14^c^	324.32 ± 16.8^a^	279.43 ± 3.34^b^	248.04 ± 12.4^b^

*Different characters in the same row data indicate significant differences (P < 0.05).*

*MT, muscular thickness; MFH, mucosal fold height.*

### Gut Microbiota

**FIGURE 3:**
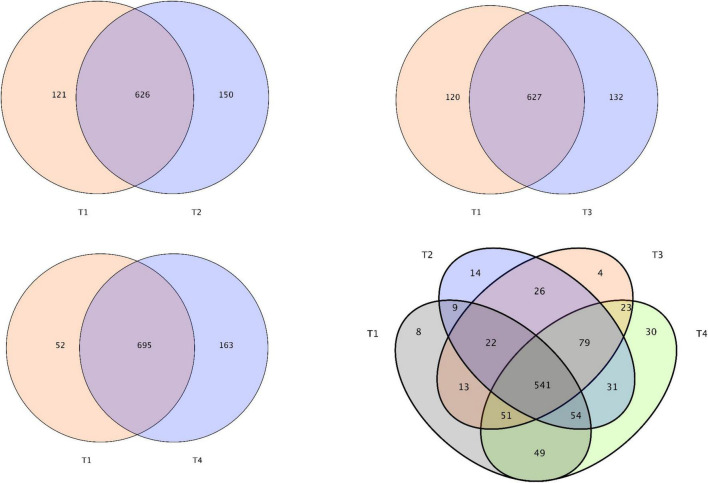
Venn diagram depicts unique and shared OTUs of gut microbiota in *E. coioides* at day 56.

**FIGURE 4:**
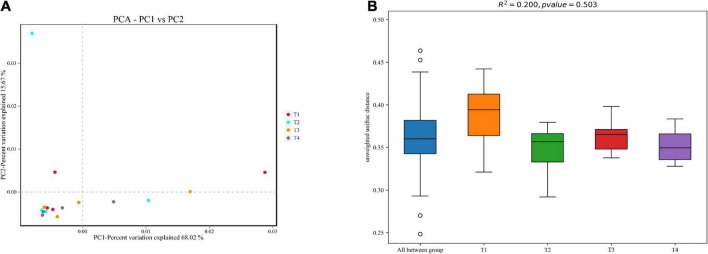
Beta diversity of gut microbiota based on unweighted UniFrac distance of *E. coioides* on day 56. **(A)** Principal component analysis (PCA) of microbial profiles from gut of *E. coioides*. **(B)** Beta (β)-diversity boxplots of microbial profiles from gut of *E. coioides*.

After removing all unqualified sequences and adaptors, a total of 732,409 effective tags were obtained from all tested samples (208,992 effective tags of T1 group, 189,104 effective tags of T2 group, 171,187 effective tags of T3 group, and 163,126 effective tags of T4 group). A total of 8096 operative taxonomical units (OTUs) were clustered from the samples with over 97% sequence similarity. The Good’s coverage index exceeding 99% across all samples suggests that the sequencing depth is adequate (Table 7). From the Venn diagrams presented in Figure 3, the number of unique OTUs in the foregut of control (T1) and experimental (T2, T3, and T4) groups were 8, 14, 4, and 30, respectively. Compared with the control group (T1), 150, 132, and 163 unique OTUs were observed in the experimental groups T2, T3, and T4, respectively (Figure 3). Compared with the control group (T1), intestinal bacterial diversity (Shannon and Simpson) and richness (Chao1 and Ace) based on OTUs showed apparent increase in groups T2 and T4 without showing any significant statistical difference (*P* > 0.05, Table 7). Principal component analysis (PCA) and β-diversity boxplots were performed to analyze the microbial similarities among all groups by unweighted UniFrac distance. A generally similar with certain varies in bacterial communities was observed between all treatments (T2, T3, and T4) and the control (T1), and the three treatments (T2, T3, and T4) demonstrated more uniform bacterial communities compared with the control (T1) (Figure 4A). In line with the PCA results, no remarkable alterations were found for the microbial β-diversity in all groups (*P* > 0.05), although certain variation was noticed in the three treatments (T2, T3, and T4) compared with the control (T1) (Figure 4B).

**TABLE 7:** Alpha (α)-diversity index of intestinal microbiota in grouper (*E. coioides*) at day 56.

	Control group	Experimental groups		
	T1	T2	T3	T4
OTUs	403.25 ± 45.31	443.50 ± 41.76	417.25 ± 53.11	521.50 ± 48.53
Chao1	430.11 ± 40.50	481.05 ± 44.41	442.57 ± 52.08	558.32 ± 41.25
Ace	422.97 ± 33.65^b^	469.90 ± 39.66^ab^	434.56 ± 51.21^ab^	555.95 ± 42.46^a^
Shannon	5.09 ± 1.36	5.15 ± 1.16	4.65 ± 1.13	5.54 ± 0.81
Simpson	0.77 ± 0.18	0.83 ± 0.09	0.79 ± 0.13	0.90 ± 0.04
Good’s coverage (%)	99.93 ± 0.02	99.89 ± 0.03	99.90 ± 0.01	99.86 ± 0.02

*Different characters in the same row data indicate significant differences (P < 0.05).*

*OUTs, operative taxonomical units.*

**FIGURE 5:**
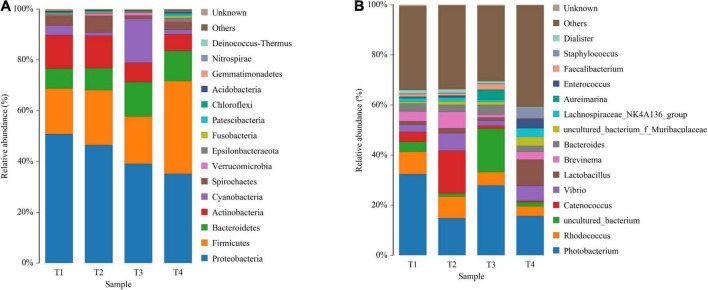
Composition and relative abundance of bacterial communities based on 16S rRNA gene sequences on day 56. Panels **(A,B)** indicate the composition and relative abundance of microbial communities at phylum and genus level, respectively.

As shown in Figure 5A, Proteobacteria, Firmicutes, Bacteroidetes, Actinobacteria, and Cyanobacteria are the most predominant phyla in all groups. Compared with the control group, experimental groups displayed the reduced relative abundance of Proteobacteria and Actinobacteria, while increased the relative abundance of Firmicutes (Figure 5A and Supplementary Table 2). At the genus level, reduced abundance of *Photobacterium* in all treatments was observed compared with the control (T1), especially in groups T2 and T4. On the other hand, *Vibrio* in experimental groups (except group T3) showed higher abundance when compared with the control group. The relative abundance of *Lactobacillus* in all experimental groups displayed a certain enhancement, especially in group T4 although no significant difference was observed (Figure 5B and Supplementary Table 2).

## Discussion

Probiotics have been widely applied in aquaculture showing abilities in promoting growth performance (Sun et al., 2013, 2014; Doan et al., 2018; Yang et al., 2019), enhancing innate immunity (Sun et al., 2013, 2014; Doan et al., 2018; Yang et al., 2019; Tang et al., 2020), and maintaining intestinal health (Yang et al., 2019; Tang et al., 2020; Zhang et al., 2020). Our previous studies have shown the efficacy of three autochthonous probiotic strains (*B. pumilus* SE5, *Psychrobacter* sp. SE6, and *B. clausii* DE5) by improving the intestinal health, innate immunity, and growth performance in grouper *E. coioides* (Sun et al., 2013, 2014; Yang et al., 2019). In our recent work, we have explored the dose-dependent effects in grouper by dietary supplementation of different levels of histamine, and the results showed that 0.3% dietary histamine can suppress the growth performance and innate immunity, and induce intestinal microbial imbalance and severe intestinal injury in grouper *E. coioides* (Liu et al., 2021). In the present study, therefore, a 56-days’ feeding trial was performed for assessing the effects of dietary supplementation with the three autochthonous probiotics on the growth, immune status, and intestinal health of grouper *E. coioides* fed with high doses of histamine (0.3%).

Evidence from several experimental studies shows that dietary supplementation with probiotics can improve growth performance and/or feed utilization in fish such as grouper (*E. coioides*; Sun et al., 2013; Yang et al., 2019), hybrid grouper (*Epinephelus fuscoguttatus* ♀ × *Epinephelus lanceolatus*; ♂ Liao et al., 2021), fingerlings great sturgeon (*Huso huso*; Rastekenari et al., 2021), rainbow trout (*Oncorhynchus mykiss*; Mohammadian et al., 2019), and Nile tilapia (*Oreochromis niloticus*; Doan et al., 2018). In line with this previous research, this study also showed that the administration of the three autochthonous probiotics can improve the growth performance and feed utilization of grouper *E. coioides* fed with 0.3% histamine, especially in fish fed with diet containing *B. pumilus* SE5 as probiotic strain (group T2), suggesting that autochthonous probiotics can improve the growth performance and feed utilization of grouper under high histamine stress. It has been demonstrated that the improvements of growth performance and feed utilization by dietary supplementation of autochthonous probiotics may be due to enhanced secretion of digestive enzymes, improved appetite, efficient liver, and gut health status as well as modulation of the gut microbiota. In this study, we speculate the observed beneficial effects may be attributed to the gut injury repairment functions of autochthonous probiotics, which will be discussed in the following paragraphs.

Stimulating the innate immune response is an important attribute of the probiotics, as they can benefit the host by improving its health status (Hoseinifar et al., 2018). In the current study, different degrees of increased activities of ACP, AKP, SOD, and T-AOC were observed in fish fed with autochthonous probiotics, suggesting that dietary probiotics can stimulate innate immune response and enhance antioxidant capacity of grouper under high histamine stress. These results are in accordance with our earlier observations that application of autochthonous probiotics, *B. pumilus* SE5 and *B. clausii* DE5, can increase the growth performance of grouper by improving the serum levels of ACP, AKP, and SOD (Sun et al., 2013). Sun et al. (2011) also reported that the dietary supplementation of *Psychrobacter* sp. SE6 can boost immune responses (increasing serum SOD activity, phagocytic activity, complement C3 and C4 levels, etc.) of *E. coioides*. To the best of our knowledge, this is the first study that demonstrates the ability of autochthonous probiotics in improving the innate immune response in fish experiencing stress due to dietary histamine stress.

Liver biochemical parameters can effectively reflect the function and health status of liver. MDA is often served as a useful bioindicator to assess the presence of oxidative damage in the liver. Additionally, the liver GPT and GOT activities specifically detect the liver injury or dysfunction (Wells et al., 1986). After 28 days of feeding, we found significant decrease in MDA levels in groupers fed with probiotic-supplemented diets, and significant improvements in GPT and GOT activities recorded only in groupers fed with diet containing *B. pumilus* SE5 as probiotics (i.e., experimental group T2) in the present study. In addition, autochthonous probiotics supplementation (experimental groups T2, T3, and T4) for 56 days showed lower MDA content and improved GPT and GOT activities in the liver compared with the control group. These results illustrate that autochthonous probiotics can alleviate liver injury caused by high dietary histamine. A similar study in shrimp (*Litopenaeus vannamei*) also demonstrated that the three beneficial bacteria (*B. pumilus* SE5, *Psychrobacter* sp. SE6, and *B. clausii* DE5) can relieve liver damage caused by high dietary soybean meal (Zhang et al., 2020).

It has been well-established that increased level of pro-inflammatory molecules (IL-1β, ITF, and FABP2) can trigger inflammation, and cause gut and local tissue injury in organisms (Wang et al., 2011; Oksaharju et al., 2013; Liu et al., 2021). The present study demonstrates that supplementation of autochthonous probiotics can alleviate inflammation caused by 0.3% dietary histamine in *E. coioides*, and these inflammation indicators reached the most ideal levels in fish fed with diets containing *B. pumilus* SE5 (group T2). A comparable result was reported by Simo-Mirabet et al. (2017) who noticed *B*acillus *amyloliquefaciens* CECT 5940 supplemented diet can activate anti-inflammatory responses in gilthead sea bream (*Sparus aurata*) after 14 weeks of feeding. To date, information on the effect of probiotics in modulating the levels of inflammatory signals (such as gut ITF, serum SAA, IL-1beta, and FABP2) in aquatic animals is less (Simo-Mirabet et al., 2017), while relatively more research has been carried out on terrestrial animals (Matsumoto et al., 2005; Wang et al., 2011; Oksaharju et al., 2013). It has been reported that feeding mice (8-weeks-old) with heat-killed *Lactobacillus casei* has decreased the amount of SAA while down-regulated remarkably pro-inflammatory cytokines such as IL-6 and IFN-γ production in lamina propria mononuclear cells (Matsumoto et al., 2005). Furthermore, similar reports have shown that *L*acticaseibacillus *rhamnosus* treatment ameliorates both intestinal and systemic inflammation by reducing plasma SAA level or suppressing intestinal ITF protein and mRNA expression in mice (Wang et al., 2011; Oksaharju et al., 2013). Oksaharju et al. (2013) hypothesized that probiotics may be conducive to modulate immune function and prevent sharp changes concerning inflammatory molecules. However, the related mechanisms in terrestrial and aquatic animals may not be completely consistent, further studies are necessary to clarify the relationship between probiotics and inflammation molecules in fish.

There are many published studies that reported that probiotics can improve the gut morphology and promote gut integrity in several aquatic animals, such as fingerlings great sturgeon (*H. huso*) (Rastekenari et al., 2021), sea cucumber (*Apostichopus japonicus*) (Zhou et al., 2021), Senegalese sole (*Solea senegalensis*) (Batista et al., 2015), and tilapia (*O. niloticus*) (Standen et al., 2013, 2016). In this study, we found significant improvements in MT and MFH, indicating that the dietary supplementation of autochthonous probiotics (*B. pumilus* SE5, *Psychrobacter* sp. SE6, or *B. clausii* DE5) can positively affect intestinal morphology of *E. coioides*. These results are in accordance with our earlier observations reporting that supplementation of probiotics (*B. pumilus* SE5, *Psychrobacter* sp. SE6, or *B. clausii* DE5) can improve intestinal villus height and MT in shrimp (*L. vannamei*) fed high soybean meal diet (Zhang et al., 2020). These similar findings may be explained by the fact that probiotics can utilize undigested carbohydrates and producing various short-chain fatty acids, such as acetic and butyric acid, which can decrease the gut pH, supply energy for enterocytes, and then improve the gut morphology.

The gut microbiota is essential to maintain intestinal immune homeostasis, which in turn modulates the gut microbial composition and health status of the host (Yang et al., 2019). In this study, the gut microbiota of *E. coioides* were changed with dietary supplementation of probiotics, which agrees with previous published studies (Tang et al., 2020). Both bacterial diversity and richness of experimental groups showed partial improvement in the present study, which may be conducive to intestinal and systemic health (Clarke et al., 2014). On the other hand, the data of β-diversity and taxonomy classification showed that the gut microbiota of fish fed with probiotics containing diets presented certain variances compared with the control, indicating that autochthonous probiotics could effectively shape the gut microbial community in *E. coioides.* A previous study has suggested that a healthier gut microbial composition can facilitate fish health via secretion of various enzymes and decreasing the gut pH by producing short-chain fatty acids (Dawood et al., 2019). Generally, common probiotics are Gram-positive bacteria and can balance the abundance of intestinal Gram-negative bacteria, thereby being beneficial to both intestine and general health of the host. In the current study, administration of autochthonous probiotics has greatly enhanced the relative abundance of Gram-positive bacteria (Phylum Firmicutes and genus *Lactobacillus*) and reduced the relative abundance of Gram-negative bacteria (Phylum Proteobacteria and genus *Photobacterium*), which suggests that probiotics can positively modulate the intestinal microbiota of groupers. Interestingly, higher abundance of genus *Vibrio* was observed in groups T2 and T4 compared with the control group. Traditionally, several *Vibrio* species were considered as opportunistic bacteria to aquatic animals. However, dietary supplementation of *Vibrio fluvialis* resulted in higher survival in rainbow trout challenged with *Aeromonas salmonicida* (Irianto and Austin, 2002). Also, combined supplementation of *Bacillus* and *Vibrio* sp. in young white shrimp showed beneficial effects on growth performance, survival as well as resistance against *Vibrio harveyi* and white spot syndrome virus (Antony et al., 2011). Recently, several reviews have summarized and discussed the potential application of *Vibrio* as a means of disease control in aquaculture at certain circumstances which contradicts the traditional knowledge (Hoseinifar et al., 2018; Butt et al., 2021). In general, these results suggest that autochthonous probiotics can positively shape intestinal microbiota and maintain intestinal homeostasis, and this may benefit the growth performance, immune function, and gut morphology of grouper *E. coioides*.

## Conclusion

This study presents evidence for the first time that the three autochthonous probiotic strains (*B. pumilus* SE5, *Psychrobacter* sp. SE6, or *B. clausii* DE5) show high efficiency in alleviating the adverse effects of (high levels of) dietary histamine by decreasing the expression of inflammatory markers and also improving the growth, innate immunity, and gut health of juvenile grouper *E. coioides*. Among the three autochthonous probiotics, *B. pumilus* SE5 exhibits the best potential to alleviate the negative effects of high levels of dietary histamine in marine fish.

## Data Availability Statement

The datasets presented in this study can be found in online repositories. The names of the repository/repositories and accession number(s) can be found in the article/Supplementary Material.

## Ethics Statement

The animal study was reviewed and approved by the Animal Care and Use Committee of Jimei University.

## Author Contributions

Z-YL: investigation and original draft. H-LY: data analysis and writing. L-HH: investigation and data analysis. WY: feed preparation and animal trial. C-XA: review and editing. Y-ZS: supervision, project administration, funding acquisition, and review and editing. All authors contributed to the article and approved the submitted version.

## Conflict of Interest

WY was employed by Xiamen Jiakang Feed Co., Ltd. The remaining authors declare that the research was conducted in the absence of any commercial or financial relationships that could be construed as a potential conflict of interest.

## Publisher’s Note

All claims expressed in this article are solely those of the authors and do not necessarily represent those of their affiliated organizations, or those of the publisher, the editors and the reviewers. Any product that may be evaluated in this article, or claim that may be made by its manufacturer, is not guaranteed or endorsed by the publisher.

## Footnotes

1
http://www.arb-silva.de/

2
www.biocloud.net
