# Supplementary material for: Autochthonous Probiotics Alleviate the Adverse Effects of Dietary Histamine in Juvenile Grouper (Epinephelus coioides)
Source: Front Microbiol. 2021 Dec 7;12:792718. doi: 10.3389/fmicb.2021.792718 (PMC8689058; doi:10.3389/fmicb.2021.792718)
Supplement: Supplementary file 1 [file Data_Sheet_1.docx]

**Supplementary data**

Table 1. Ingredients and proximate nutrient composition of the basal diet (% dry matter)

| Ingredients | Content (%) |
| --- | --- |
| White fish meal^1^ | 43.0 |
| Shrimp head meal | 3.0 |
| Soybean meal | 16.88 |
| Vital Wheat Gluten | 7.00 |
| Fish oil^2^ | 2.5 |
| Soybean oil | 2.5 |
| Lecithin | 2.0 |
| Wheat meal | 20.0 |
| Choline chloride^3^ | 0.5 |
| Vitamin premix^4^ | 0.5 |
| Mineral premix^5^ | 0.5 |
| Ca(H_2_PO_4_)_2_ | 1.5 |
| Antifungal agent | 0.10 |
| Antioxidant | 0.02 |
| *Proximate nutrients composition* |  |
| Crude protein | 45.85 |
| Crude fat | 12.16 |
| Crude ash | 9.82 |

^1^White fish meal was obtained from Jiakang Feed Co. Ltd., Xiamen, China, imported from Peru (crude protein 68.34%, crude lipid 9.06%).

^2^Fish oil were obtained from Jiakang Feed Co. Ltd., Xiamen, China.

^3^Cholesterol was produced by Baiwei Biotechnology Holdings Co., Ltd, Hebei, China, which is extracted from pig, cattle or sheep brain and the minimum level is higher than 95%.

^4^Vitamin premix (mg kg^-1^ diet): vitamin A, 15; vitamin D3, 15; vitamin E, 75; vitamin K3, 50; vitamin B1, 50; vitamin B2, 75; vitamin B6, 75; vitamin B12, 0.3; nicotinic acid, 200; inositol, 350; *D*-calcium pantothenate, 200; folic acid, 9; *D*-biotin, 0.5.

^5^Mineral premix (mg kg^-1^ diet): FeSO_4_·7H_2_O, 278; CuSO_4_·5H_2_O, 41; ZnSO_4_·7H_2_O, 463; MnSO_4_·4H_2_O, 57; MgSO_4_·7H_2_O, 2009; CoSO_4_·7H_2_O, 3; Na_2_SeO_3_ 0.6, Ca (IO_3_)_2_, 5.

Table S2MetaStat analysis of the abundance of intestinal bacterial phylaand genera (×10^-4^)at day 56

|  | Groups | | | |
| --- | --- | --- | --- | --- |
|  | T1 | T2 | T3 | T4 |
| **Phylum** |  |  |  |  |
| Proteobacteria | 4725.33±1619.45 | 4304.86±1427.91 | 3839.85±1505.65 | 3421.06±864.67 |
| Firmicutes | 1935.71±668.07 | 2383.81±974.68 | 1997.79±1193.42 | 3710.44±701.56 |
| Bacteroidetes | 1111.80±33.49 | 986.53±428.77 | 1524.32±708.22 | 1241.53±379.39 |
| Actinobacteria | 1408.23±436.17^ab^ | 1683.84±125.45^a^ | 741.37±234.00 ^b^ | 666.18±233.99 ^b^ |
| Cyanobacteria | 398.92±121.21 | 127.93±35.61 | 239.10±17.99 | 190.55±93.91 |
| **Genus** |  |  |  |  |
| *Photobacterium* | 2757.05±2186.63 | 1327.94±1196.06 | 2670.23±1671.78 | 1461.168±1000.98 |
| *Rhodococcus* | 968.05±301.78 | 899.42±272.79 | 505.62±169.57 | 404.58±143.29 |
| *uncultured_bacterium* | 405.26±122.89 | 129.25±35.56 | 1528.32±882.73 | 172.76±84.48 |
| *Catenococcus* | 431.53±327.84 | 1438.40±1432.80 | 123.64±60.57 | 48.90±21.80 |
| *Vibrio* | 300.17±129.35 | 686.11±419.02 | 213.16±123.97 | 617.85±388.75 |
| *Lactobacillus* | 148.67±48.25 | 226.18±110.79 | 137.48±114.00 | 1105.22±1018.37 |
| *Brevinema* | 418.21±413.85 | 581.60±553.71 | 143.27±112.93 | 274.30±270.97 |
| *Bacteroides* | 334.47±125.89 | 313.27±142.17 | 428.96±305.96 | 242.28±64.81 |
| *uncultured_bacterium_f_Muribaculaceae* | 85.34±38.89 | 161.50±87.23 | 136.90±107.11 | 385.84±185.11 |
| *Lachnospiraceae_NK4A136_group* | 134.87±53.49 | 156.58±65.33 | 84.15±43.22 | 384.77±285.53 |
